# Dr. John Phelps Shumway

**Published:** 1912-05

**Authors:** 


Dr. John Phelps Shumway of Los Angeles, died March 17, 1912, aged 75. He was graduated at Albany Medical College, 1860 and had practiced at Geddes and Syracuse.

